# Lectures on the Diseases of Infancy and Childhood

**Published:** 1853

**Authors:** 


Art. XVI. — *Lectures on the Diseases of Infancy and Childhood.* By Charles West, M. D., Fellow of the Royal College of Physicians to the Hospital for Sick Children; Physician-Accoucher to, and Lecturer on Midwifery at, Saint Bartholomew's Hospital. Second American, from the Second and Enlarged London Edition. Philadelphia: Blanchard & Lea. 1854.

We have in the volume of Lectures of which we have given above the title, a practical work, a new edition of which renders it available to all our readers. It is founded upon the observations of the author at the Lambeth Infirmary for Children, continued through ten years. We can find space for only a few extracts from its instructive pages. In reference to the frequency of convulsions in children, the author remarks :—

"The grand reason of their frequency is no doubt to be found in the *predominance of the spinal over the cerebral system in earl*y *life.* In the adult the controling power of the brain checks the display of those reflex movements which become at once evident if disease heighten the excitability of the spinal cord, or cut off the influence of the brain from the paralysed limb, or even if sleep suspend that influence for the season. When the child is born the brain is but imperfectly developed, its functions are most humble, and convulsions are then so frequent that they are computed to occasion 73.3 per cent. of all deaths which take place during the first year of existence, from diseases of the nervous system. In the next two years the brain more than doubles its weight, and deaths from convulsions sink to just a third of their former frequency. In proportion as the brain increases in size, and its structure acquires perfection, and its higher functions become displayed, convulsions grow less and less frequent, until from the 10th to the 15th year they cause less than 3 per cent., and above 15 less than 1 per cent., of the deaths from diseases of the nervous system.

But a little observation will show you, that though convulsions are often the immediate cause of death, yet this fatal event is rare during childhood in comparison with those instances in which they pass off without any serious result; and that in proportion to their frequency they less often betoken serious disease of the brain in the child than in the adult, while any cause which greatly excites the spinal system may be attended by them. The disturbance of the spinal system, which ushers in fever in the adult, shows itself by shivering. In the child, the same disturbance often shows itself not by shivering but by convulsions; or convulsions may be induced by a constipated state of the bowels, by the presence of worms in the intestinal canal, or of a calculus in the kidney, or by the pressure of a tooth upon the swollen gum. Hence your first duty is, in every case, to ascertain where is the seat of the irritation which excited the nervous system to this tumultuous reaction. If the fits come on in an advanced stage of some serious disease, they are probably only the indications that death is busy at the centre of vitality; if they attack a child laboring under hooping-cough, they point to a congested state of the brain, the consequence of the impeded circulation through the lungs; if they occur in a child apparently in perfect health, they probably indicate that the stomach has been over-loaded, or that some indigestible article of food has been taken; or, if that be certainly not the case, one of the eruptive fevers is perhaps about to come on; most likely either small-pox or scarlatina."

Dr. West thus speaks of a condition of the lungs sometimes found in infants at birth which leads to early death —*atelektasis*, or imperfect expansion of the lungs, first clearly described by Dr. Jorg, of Leipsic:—

"The *causes* of this condition are not clearly made out. Dr. Jorg has attributed great importance to precipitate labour as a frequent cause of its occurrence, and has suggested a somewhat fanciful theory to explain its mode of production. He conceives that one grand use of the uterine contractions is gradually to enfeeble the circulation through the placenta, and thus to induce in the fœtus that *besoin de respirer* which shall excite the complete establishment of respiration immediately on its birth. If, however, by the very rapid course of labor, the child should be born while the fœtal circulation is still going on with unimpaired vigor, the want of air will not be experienced by the child, and its attempts to breathe will be feeble and imperfect. It is probably better, instead of indulging in speculations of this sort, to content ourselves with the simple statement that when, from any cause whatever, the establishment of respiration at all has been attended with difficulty, there is a very great probability that its establishment will never be complete, but that some lobules only will receive the air, while it will not penetrate into other parts of the lung. The probability of this occurring, too, will be still greater if the children be weakly, or ill-nourished when born, or if they be exposed soon after birth to cold or other unfavorable hygienic influences, such as are calculated to interfere with the due performance of respiration.

Cases in which this condition of the lungs exists usually present the history of the child having been apparently still-born; and, though resuscitated after a time, yet still continuing unable to utter a strong and loud cry like that of other children. Even after breathing has gone on for some time, such children usually appear feeble; and though they may have attained the full term of fœtal life, yet they can scarcely suck, although they often make the effort. An infant thus affected sleeps even more than new-born infants usually do; its voice is very feeble, and rather a whimper than a cry; and the chest is seen to be very little, if at all, dilated by the respiratory movements. The temperature falls, the skin becomes pale, and the lips grow livid, and often slight twitching is observed in the course of a few hours about the muscles of the face. The difficulty in sucking increases, the voice grows weaker and more whimpering, or even altogether inaudible, while respiration is attended with a slight *rale*, or an occasional cough; and the convulsive movements return more frequently, and are no longer confined to the face, but affect also the muscles of the extremities. Any sudden movement, suffices to bring on these convulsive seizures; but even while perfectly still the child's condition is not uniforn, but it will suddenly become convulsed; and during this seizure the respiration will be extremely difficult, and death will seem momentarily impending. In a few minutes, however, all this disturbance ceases; and the extreme weakness of the child, its inability to suck, its feeble voice, and its frequent and imperfect inspirations, are the only abiding indications of the serious disorder from which it suffers. But the other *symptoms* return again and again, till at length, after the lapse of a few days, or a few weeks the infant dies.

But I will relate a case which may serve to impress these characteristics on your memory. A little boy, three weeks old, was brought to me at the Children's Infirmary, on March 13, 1846. He was puny, emaciated, with a cold surface, and bloodless conjunctiva. His face, which was wizened like that of an old man, was occasionally distorted by slight convulsive twitches; and these fits, as the mother termed them, were, according to her account, sometimes much more severe. The abdomen was tympanitic, and it alone was seen to move during respiration, there being hardly any lateral expansion of the chest. The ear applied to the chest heard but little air entering; and the cry was a stifled whimper, in which none of the inspiratory sound, the *reprise* of French writers, was distinguishable. The child sucked with difficulty, and had wasted ever since its birth, though no diarrhœa existed, but the bowels, on the contrary, showed a tendency to constipation.

The chest was rubbed twice a day with a stimulating liniment, and a mixture was given containing some ammonia and the compound tincture of bark. Under this treatment the child appeared to improve; it began to breathe less rapidly and in a less labored manner; and its cry became louder. The parents, however, were miserably destitute, the mother in an ill state of health, so that her milk afforded but a very imperfect sustenance for the child. From the beginning of April he grew less well, and began to have occasional attacks of general convulsions, in one of which he died on April 26, 1846.

On examining the body, large portions of both lungs presented the appearance which I have described as characteristic of their imperfect expansion; but inflation restored them to a crepitant state. Some patches, however, though they admitted air and assumed the same color as the rest of the lung, yet could not by any effort be dilated so completely as to rise to a level with the surrounding tissue. The foramen ovale was open, the margin of the valve for fully half its circumference not being adherent, although the valve was sufficient large for its closure. The ductus arteriosus also was quite permeable, although of considerable less calibre than during fœtal life."

